# Editorial

**Published:** 1858-03

**Authors:** 


### Editorial.

### PECULIARITIES OF POST OFFICE DEPARTMENT.

In a great many instances where we have been sending the *Register* regularly for the past year and a half, it has uniformly failed to reach the subscribers till a bill was sent with it; that one is always sure to be a *dead shot*: for we are forthwith notified that, that is the only number ever received, or, perhaps, the only one since the first. Now this would seem as though the Post Masters treat our subscribers *shabbily*.

Why is it they do not send till one comes along with a bill? then they seem to take extra care to secure the safe delivery of that one. Well, it is probably out of kindness to the poor *printers*.

This has occurred to us from the fact, that three-fourths of all the numbers returned, are those containing bills, and generally with the remark that "that is the only one received," or possibly with one exception Prompt paying subscribers, seldom, if ever, fail to get their numbers regularly. But we are annoyed almost daily by non-paying subscribers about missing numbers.

We hope that no one will continue to receive the *Register* who does not want it, but return it promptly.

### CHEOPLASTY.

The subject has been somewhat prominently before the profession in this city recently. The time has not been long enough, perhaps, for many or even any, to arrive at a definite conclusion, in regard to its merits from actual experiment.

Several months ago we made some hurried experiments by this method, put up ten or twelve pieces. They were all made with the idea, that it was necessary, for strength, to make the plates two or three times as thick as gold plate. The plates we have seen recently were not more than 24 to 26 stubes guage; and yet they seemed to possess sufficient strength for ordinary cases. In our first efforts, doubtless the thickness of the plates was a material mistake; all of our pieces were clumsy in the mouth. They were made during a press of other business, and consequently did not receive that attention and care, that seems necessary to insure success. In none of these cases was there any special difficulty experienced by change of the metal, though none of them were worn more than a month or six weeks.

Upon the integrity of the metal, however, there is a difference of opinion. But the testimony of those who have used it longest and most, is, that it seems to answer the requirements fully in this respect.

From what we know from others, there is little doubt, that under ordinary circumstances, it will stand the test so far as destructability is concerned. We do not think that sufficient time has elapsed, to enable any one to determine how it may be affected by peculiar circumstances, which are sometimes found; as for instance in cases of ptyalism; in fully marked cases of dyspepsia; where there is frequent acid eructations; and in cases where the secretions of the mouth are changed to an acid condition. Also in a mouth where there are a number of teeth remaining, in which there are gold fillings, that may come almost or altogether in contact with the plate. How this metal will be affected by these circumstances time alone will demonstrate. But it can be ascertained, if all those who are using it, will observe all such cases, and note every change.

This work can be made as perfect in adaptation, as any other style. The metal is perfectly adapted to the teeth, and no interstices afford lodgement for food or any other foreign substance. Any desired form may be given to the pieces, for the comfort and convenience of the contiguous parts.

---

### MISSISSIPPI VALLEY ASSOCIATION.

This, the oldest dental association in existence, held its annual sessions on the 17th, 18th, and 19th of February, in the Ohio College of Dental Surgery.

There was about the usual number in attendance, though we missed many we hoped to see there. But new members came in to take their places, but it made us sad to miss some of our old friends, with whom we have heretofore spent so many pleasant hours, and worked so harmoniously.

The discussions, which are reported in the present number of the *Register*, were spirited and very interesting ; and in many particulars very instructive.

One thing is very apparent in all these meetings and discussions, and that is, that those who have been for a long time conferring together, and consulting upon topics of practice, perform their operations in very nearly the same manner, and obtain almost uniform results.

Every one who reads the report of the discussions may consider that he does not receive half as much as he would have received had he been there. Though the discussions are fully reported, all can not be told that was said ; the manner too is lost ; and probably new and valuable suggestions would have been brought forth, had a greater number been there. We hope that in future, old scores will be paid by prompt attendance.

---

### COMMITTEE ON IRREGULARITY.

It will be seen by reference to the minutes of the Mississippi Valley Association, that a standing committee on irregularity of the teeth was appointed. This we think a decidedly good move. This is a part of operative dentistry with which the profession as a whole, is probably less familiar than any other ; dentists have long hesitated to take hold of it ; only one here and one there has felt himself adequate to the task, except in some simple case.

The members of this committee have long made this branch a study, and we think from their familiarity with the subject, they will set it forth in a desiable form. We take it to be the duty of this committee, to give any principles—that have not been hitherto detailed—involved in the proper performance of this operation. It is their duty to seek every thing new in regard to it—gathering from all available sources. It is also the duty of the profession to aid them in every way possible ; giving them cases, models, drawings, and definite descriptions, and we are certain the committee would not slight good suggestions. The committee consists of Drs. James Taylor, H. R. Smith and C. Bonsal, who are ready to receive any thing that may be communicated on the subject. The committee is expected to prepare matter for the June number of the *Register*, and if so they should have material to work upon immediately.

---

### AMERICAN DENTAL REVIEW.

Our old friend, Dr. A. M. Leslie, once connected with the editorial department of the *Register*, has started a journal at St. Louis, with the above title. It is to be published quarterly, each number containing forty-eight pages, at fifty cents per annum, in advance. The first number makes a fine appearance, and is well filled, both in its original and select departments. We have no room, in our present number, or we would borrow from its original articles. We are happy to add it to our list of exchanges. You can all afford to take it as it comes cheap ; indeed, if the remaining numbers equal the first, it will hardly be honest for you to read all of them for merely the small pittance of fifty cents. The worst fault of the *Review* is that it is too much of a JOURNAL to be afforded at the price of an ALMANAC.

### NEW IMPRESSION CUP.

This instrument, which was invented and made by Dr. Fairbank, of St. Louis, has for its object, in addition to holding the wax, chilling or hardening while in the mouth ; so that the impression will not be injured by its removal from the mouth. Every one will recognize this as an important item, if it can be accomplished. In the use of this instrument it is perfectly done.

It is a double cup, formed of two plates united at their edges all round ; with a little space between them. To this are attached two tubes ; through one cold water is made to pass from a rubber holder,—of about one pint capacity—and tube. The water passes off through the other tube into a vessel conveniently arranged to receive.

Will the *Dental Review* give this instrument an name ?

CAMTETER—ZERANTER—UGH !

### A PROPOSED VOYAGE.

The *American Dental Review* proposes an *extension* of the American Dental Convention, by taking it on board a steamboat, down the Ohio river, up the Mississippi, and then by rail across the prairies of the West. Well, if the Ohio should be August-like, the excursion would resemble a "perilous voyage on the disastrous waters of the raging canawl ;" and if the steamer should stick on a sand bar, theConvention might find it difficult to adjourn. But even if the river should be "like herself," there is this great objection to the plan : it would be making the pursuit of scienec a secondary object of the meeting, or at least giving it a secondary place.

### PERSIAN BALM.

This is a liquid soap neatly put up by Dr. S. S. Blodget, of Ogdensburg, N. York.

Many things are claimed for it, but we refer to it only as a preparation for the teeth.

When any thing of the saponaceous character is desired, we know of nothing so good as this. It is very pleasant in the mouth, forms a beautiful lather, and cleanses the teeth as well if not better than any soap we are familiar with. We have been using it for several months, and are better pleased with it the longer we use it, and we don't intend to be without it. Try it. It may be obtained at the dental depots.

### THE DENTAL CONVENTION OF NORTHERN OHIO.

This association meets in Cleveland on the first Tuesday of May next.

This association sprang into a vigorous existence in November last, and bids fair to "leave some of its predecessors behind."

To all who possibly can, we say go to Cleveland ; we can assure you, you will be glad you did.

### PHOSPHATE OF LIME LOZENGES.

Those who wish to prescribe the sub-phosphate of lime, for difficult or imperfect dentition, can obtain it neatly prepared in the form of lozenges, by addressing A. Packham, Cincinnati, Ohio, or Jno. T. Toland. They are prepared by Mr. P., whose integrity is sufficient guaranty for their purity and genuineness.

*The Quarterly Journal of Dental Science, London; Walton and Maberly, Upper Gower Street, and Ivy Lane. Paternoster Row.* Price, five shillings.

Vol. 1, No. 4 of the *Quarterly Journal* is before us. It is well printed, on heavy paper, contains 126 pages, and is filled with a variety of interesting matter. We have not had time to examine all its articles with that care which we intend to bestow on them. By whom it is edited we know not, nor does it matter as long as it is well edited. We hope to receive this valuable auxiliary with regularity hereafter. We have sent the *Register* to its address regularly, yet but one number, it seems, has been received. The *Quarterly* may have been sent with the same regularity, yet we have received but the one number. We can not refrain from noticing, in this connection, the handsome compliment to our contributors. The editor says, "It has hitherto been a *fault*—and we feel assured our professional brethren on the other side of the Atlantic will forgive us for stating the fact—that the dental papers in American publications have been too verbose, and required the pruning knife of the editor to cut them into a really practical shape." And farther: "To this point, it is evident, the editors of the *Dental Register* have directed their attention; for we find, amongst the original communications some valuable practical papers, &c."

Now, in the number referred to, our contributors appear just as they wrote. The pruning knife might have been applied, perhaps, but it was not. As soon as we have room we will lay before our readers some selections from the "*Quarterly Journal*; and in the mean time, you who have the five shillings, sterling, to spare, would do yourselves a kindness to subscribe for it.

---

DR. RUFUS SOMERBY.—On the twenty-sixth of January last, the City of Louisville, Ky., sustained an irreparable loss in the sudden death of one of her oldest and most respected citizens, and many a heart felt a pang of poignant grief, as the sad tidings spread upon the street that Dr. Somerby was "no more."

There is at all times a sadness in those words—a sadness of regret when childhood and youth are cut down by the hand of death, as the lights are sometimes extinguished in the house of feasting, and the sudden mantle of darkness falls upon its joyous inmates—a sober sadness, not unmixed with a contented resignation, when the very aged, whose tottering steps have hardly sustained them in the road of life, fall quietly to rest in the pathway. And as we contemplate the sleeper who has thrown from his feeble shoulders the burden of life, we say, with faltering accents perhaps, but without wronging the rest of the wearied sleeper by one wish for his return, "he is no more."

How different are our feelings when at the bedside of him whose strength is still unimpaired by life's journey, and who with vigorous step and calm assurance in the rectitude of his "walk in life," has marched in the foremost rank of those whose onward progress is the result of manly energy and independent exertion for the good of their fellow-men! Then comes over us a feeling of awe, and we weep bitter tears that the strong man is thus cut off in his strength, when he could look from the height to which he had attained, and review his toilsome pathway with satisfaction at his success; and the higher pleasure derived from the aid which he had extended to his fellow-travelers upon the hard and weary road.

He, whose death we are thus called upon to lament, was born in the town of Newburyport, in Massachusetts, on the 9th of January, 1791. He was of English descent, though his father, like most of his countrymen, became thoroughly filled with the spirit of freedom, and served actively in the war of the revolution, and though but a youth at the time, was detailed for guard duty around the scaffold upon which the intrepid Andre paid the penalty of death.

Dr. Somerby spent his boyhood in his native town, and, as is common with boys upon the sea-coast, became imbued with a strong desire to follow the sea. Although he gained sufficient knowledge of the art of navigation to understand the management of a ship, his sailor-life was confined to a voyage to Europe, when the war of 1812 so interfered with the commerce of New England, that he abandoned the sea forever.

Being in delicate health, he left home and established himself in some mercantile pursuit at Alexandria, D. C., and while thus engaged, he received the offer of a lieutenantcy in a Spanish privateer; this, however, he promptly refused.

After a short residence there, he removed to Georgetown, D. C., where, meeting with Dr. Durroux, a prominent dentist, he concluded to embrace the profession of Dentistry, and for that purpose placed himself under the tutelage of his new friend. Having gained a thorough knowledge of his profession, he practiced it most successfully both at the South and West, residing in several different places, New Orleans being the most important, and there he remained in full practice for seven years. The climate of the South began, however, to affect his health, and he was forced to leave for the West, and settled in Cincinnati in 1828, where he enjoyed a wide reputation, and stood deservedly in the front rank among his professional brethren.

Louisville, however, became his home, and here he spent the last twenty-three years of his useful life. Here he was known, appreciated, and respected, and here, as around a home, his interests and affections centered.

Firm in his sense of right, and just in his dealings with others, Dr. Somerby was always the kind and courteous gentleman; and this courtesy was not alone the courtesy of manner, but the true politeness of the heart.

Interested in the progress of society, he had none of that rashness which seizes upon everything that claims to be good, but the goodness of which consists only in its novelty. Still, though conservative, he was never one to hinder a project which was intrinsically good.

He possessed clear and elevated views upon the subject of education, and in the youthful freshness of his heart, took an earnest interest in the progress of the young.

In closing this brief article, we would add that Dr. Somerby stood at the head of his profession, and was chosen President of the Mississippi Valley Association of Dental Surgeons in September, 1851. Thus enjoying the respect of the community in which he lived, and beloved by all who knew him, we leave his name and memory to be cherished by the hearts of the many who were wont to meet his sympathy, and receive his aid in the troubles that bestrew life's pathway—and to that smaller circle whose central light is gone, and whose hearts are filled with a grief that will not admit of a profane touch from a stranger hand. G.

---

At a meeting of the dental profession of Louisville, on the 26th of January, 1858, at the residence of Dr. Samuel Griffith, called upon receiving intelligence of the death of Dr. Rufus Somerby, Dr. N. Clute was called to the chair, and Dr. J. A. McClelland appointed secretary.

The following preamble and resolutions were unanimously adopted:

It having pleased an all-wise Providence to call from the scene of his earthly labors and the field of his usefulness, our friend and brother, Dr. Rufus Somerby, so widely known and appreciated for his talents, his rare professional skill, and his many admirable qualities of heart. Therefore,

*Resolved*, That we have received with profound regret, the intelligence of the decease of our late associate.

*Resolved*, That in the death of Dr. Somerby our profession has lost a bright ornament, dental science a zealous, intelligent advocate, and our city an excellent and useful citizen.

*Resolved*, That with heartfelt gratitude for the services rendered by Dr. Somerby to the profession of which he was a distinguished member, and to the community in which he lived, we will cherish his memory with affectionate regard.

*Resolved*, That we tender to the bereaved family of the deceased our warmest sympathy, and that the Secretary be directed to transmit to them a copy of these resolutions, and furnish a copy to each of the dental periodicals, and to the daily papers of the city for publication.

LOUISVILLE, FEB., 1858. J. A. McCLELLAND, Secretary.

